# Risk factors for lymph node metastasis of soft tissue sarcomas of the head, neck, and extremities, and the clinical significance of negative lymph node dissection

**DOI:** 10.1186/s13018-022-03050-3

**Published:** 2022-03-18

**Authors:** Qi-Kun Liu, Xiao-Jun Yu, Ying-Guang Wang, Rui Lu, Shan-Xi Wang, Hao-Ran Xu, Hao Kang

**Affiliations:** Department of Orthopaedics, Tongji Hospital, Tongji Medical College, Huazhong University of Science and Technology, 1095 Jiefang Avenue, Qiaokou District, Wuhan, 430030 Hubei Province China

**Keywords:** Soft tissue sarcoma, Lymph node metastasis, Risk factors, Prognosis, Negative lymph node dissection

## Abstract

**Background:**

This study sought to define the risk factors for lymph node metastasis (LNM) of soft tissue sarcomas (STS) of the head, neck, and extremities, and the clinical significance of negative lymph node dissection (NLND).

**Methods:**

STS patient data in the Surveillance, Epidemiology, and End Results (SEER) database from 1988 to 2015 were extracted and pooled. Logistics regression analysis was used to identify risk factors for LNM, Cox proportional hazards and Fine–Grey’s models were used for survival analysis, and Propensity score matching analysis (PSM) was used to assess the impact of NLND on patient prognosis.

**Results:**

A total of 3276 patients were enrolled in the study, of whom 283 (8.6%) developed LNM. Rhabdomyosarcoma had the highest rate of LNM (25.3%), followed by clear cell sarcoma (16.8%) and epithelioid sarcoma (12.4%), while leiomyosarcoma had the lowest rate of LNM (1.3%). Sex, tumor size, grade, histology, and site were significantly associated with LNM. For specific histologic subtypes of STS, NLND significantly improves overall survival (HR: 0.718, 95%CI 0.535–0.962; *P* = 0.026) and cancer-specific survival (HR: 0.699, 95%CI 0.506–0.967; *P* = 0.031) and reduces cancer-specific mortality (Gray’s test, *P* = 0.017). However, NLND did not improve overall survival (*P* = 0.46) or reduce cancer-specific mortality (Gray’s test, *P* = 0.772) of patients with leiomyosarcoma.

**Conclusions:**

Histology is an independent risk factor for LNM in STS of the head, neck, and extremities. Prophylactic NLND treatment was necessary and had a clinical benefit for patients with STS who were at high risk for LNM but had no significant impact on the prognosis of patients with leiomyosarcoma.

**Supplementary Information:**

The online version contains supplementary material available at 10.1186/s13018-022-03050-3.

## Introduction

Soft tissue sarcomas (STS) are rare heterogeneous solid tumors of mesenchymal cell origin of which more than 50 different histologic subtypes have been identified to date [1]. Common subtypes of STS include malignant fibrous histiocytoma, liposarcoma, and leiomyosarcoma. The extremities (30.7%), truncal or visceral locations (50.4%), retroperitoneum (11.7%), and head or neck (7.2%) are the most common primary sites [2]. These mesenchymal tumors have a propensity for hematogenous metastasis with distant metastasis rates ranging from 12 to 37.7% [3, 4]. Lung and bone metastasis is common [4, 5], while lymph node metastasis (LNM) is relatively rare in most STS [6], with an incidence of 0.9–6% [3, 7–10]. The rate of LNM varies greatly by sarcoma type. A retrospective study by Keung et al. involving 89,870 extremity/trunk STS found that small cell sarcoma (19%), clear cell sarcoma (16%), epithelioid (13%), and angiosarcoma (6%) were the subtypes with the highest incidence of LNM [8]. Sawamura et al. found that the STS with the highest rate of LNM were clear cell sarcoma (38%), rhabdomyosarcoma (37%), epithelioid sarcoma (30%), angiosarcoma (20%), and Ewing’s sarcoma of soft tissue (16%) [9]. Some studies have classified clear cell sarcoma, rhabdomyosarcoma, epithelioid sarcoma, and angiosarcoma as subtypes with a high risk of LNM [11]. Based on these findings, six STS subtypes with a high risk of LNM were selected for analysis in this study.

It is generally believed that LNM is associated with a poorer prognosis [3, 10, 11]. Crettenand et al. found that LNM reduces both overall (median survival: 15.1 vs. 73.9 months, respectively; *p* = 0.002) and disease-free survival (median disease-free survival: 8.0 vs. 33.0 months, respectively; *p* = 0.006) [12]. Metastatic disease is also the first sign of many occult malignancies, and metastases can cause complications that impair quality of life [13, 14]. The 8th edition of the American Joint Committee on Cancer (AJCC) staging system defines lymph node involvement as stage IV disease in sarcomas of the trunk and extremities. Thus, identifying patients who are at risk for LNM and defining LNM-specific risk factors is important for clinical decision-making and patient prognosis. However, identifying these patients has always been a challenge. Identifying at-risk patients has been a challenge, however, and the prognostic factors of STS patients without LNM who have undergone surgical treatment are not well-defined. At present, there are no studies on the prognostic impact of NLND treatment on patients with STS of the head, neck, and extremities. Thus, the present study sought to identify the risk factors for LNM in STS patients with six histological subtypes, the prognostic factors of patients without LNM, and the impact of NLND treatment on patient prognosis.

## Materials and methods

### Data sources and patient selection

**Fig. 1:**
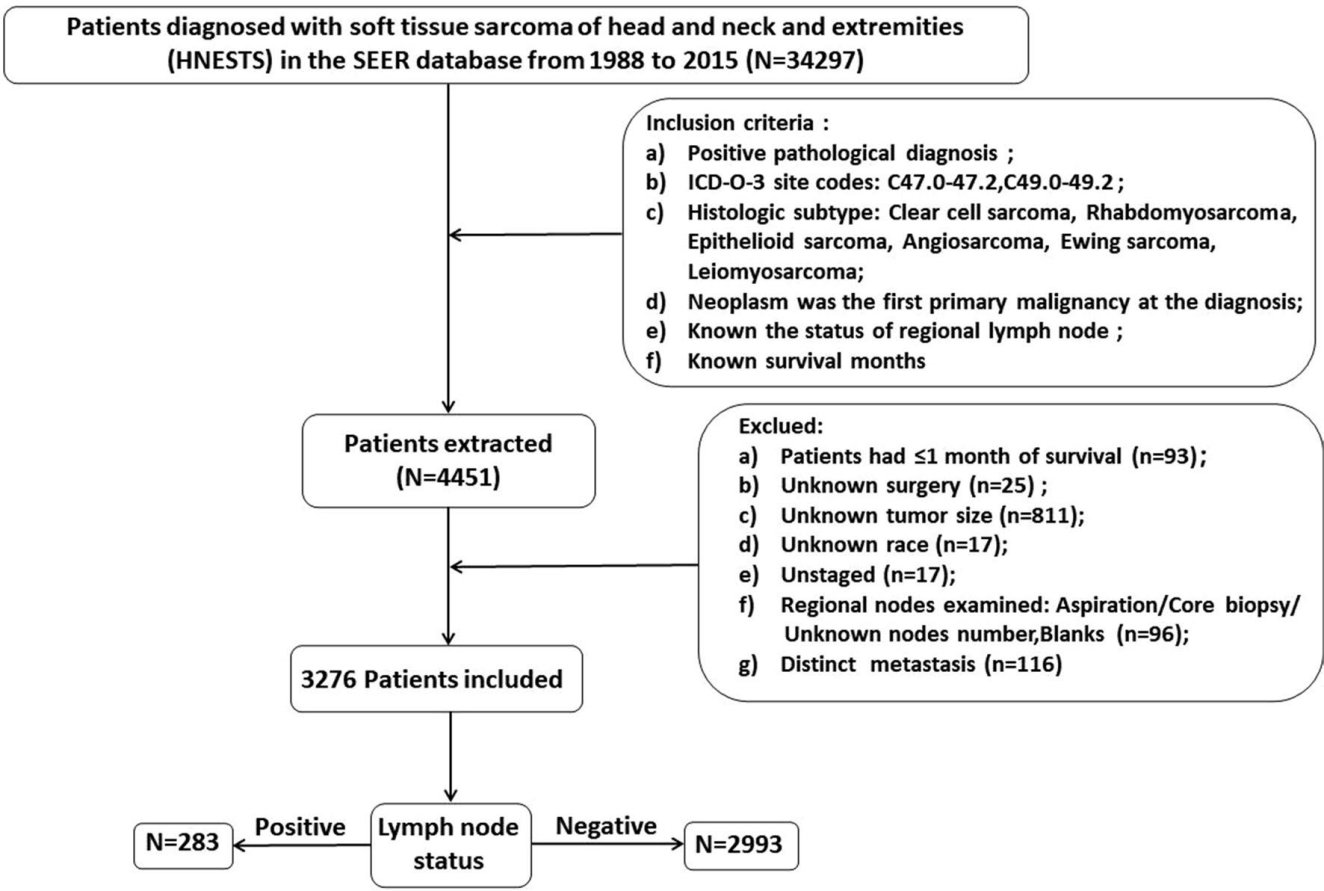
Flowchart of the selection of patients with STS of the head, neck, and extremities from the Surveillance, Epidemiology, and End Results database

Clinical data for this retrospective study was obtained from the Surveillance Epidemiology and End Results (SEER) database. The SEER database is free and publicly accessible, and individual consent for this retrospective analysis was waived because the patient information is anonymous. The study was conducted according to the Declaration of Helsinki (as revised in 2013). Patient data were downloaded using the SEER*Stat software (version 8.3.6). The final study population was selected based on the following inclusion criteria: (1) diagnosis based on positive pathology, (2) tumor sites in the head, neck, and extremities, (3) histological codes: 8890-1, 8893-6, 8900-8902, 8910, 8912, 8920, 9120, 9130, 9133, 9150, 9170, 9260, 9364, 9473, 8804, 8005, 9044, 4) specified tumor was the first primary tumor at the time of diagnosis, and 5) available data on lymph node status and survival month. The detailed inclusion and exclusion process is shown in Fig. 1.

### Variables and outcomes

**Fig. 2:**
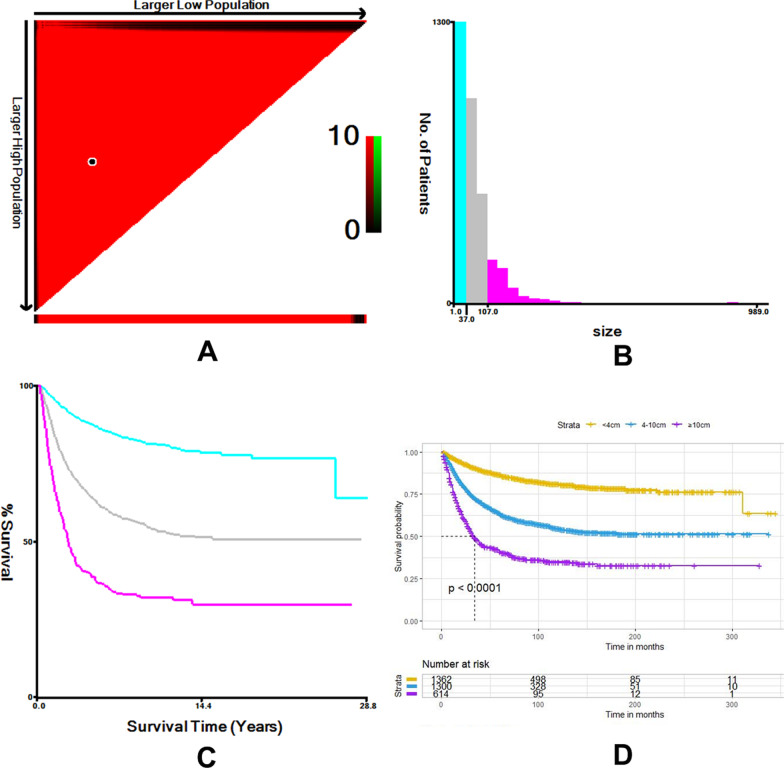
Optimal tumor size cut-off values according to cancer-specific survival by X-tile analysis. X-tile software was used to calculate the cut-off value of tumor size (**A** and **B**). The cohort was divided into low (purple), medium (gray), and high (blue) survival groups according to the cut-off value for tumor size (**C**). The cut-off value for tumor size was validated by Kaplan–Meier curve (**D**)

The following information was collected for each patient: (1) demographic variables including age, sex, race, marital status, and year of diagnosis, (2) oncological variables including tumor size, site, laterality, stage, grade, lymph node status, and histology, and (3) therapeutic variables including surgery and lymph node dissection. Prior research has shown that the oncologic features of STS patients differ by age group. For this study, age groups included those < 19 years of age (children and adolescents) and ≥ 19 years of age (adults). Since SEER began including information on lymph node dissection in 1988, data were only collected for cases recorded from 1988 to 2015. The years of diagnosis were divided into two categories: 1988–2005 and 2006–2015. Tumor histological subtypes were determined using the “ICD-O-3 Hist/Behav” field in the SEER database. Six STS types with the following histological codes were included in the study: 8890-1, 8893-6, 8900-8902, 8910, 8912, 8920, 9120, 9130, 9133, 9150, 9170, 9260, 9364, 9473, 8804, 8005, and 9044. Tumor sites were divided into three groups according to the “Primary Site—labeled” field in the SEER database. Staging information was obtained according to the SEER historic stage A (1973–2015) field. Lymph node status information was obtained from the “EOD 10-nodes (1988–2003)” and “CS-lymph nodes (2004–2015)” fields for different periods, and further divided into negative (N0) and positive (N1). Regional lymph node dissection information was obtained from the “Regional nodes examined (1988+)” fields. Lymph node “dissection” was defined as the removal of most or all of the nodes in the lymph node chains that drain the area around the primary tumor, include lymphadenectomy, radical node dissection, and lymph node stripping. Patients with a history of lymph node aspiration or core biopsy, sentinel node procedures, or an uncertain number of removed lymph nodes were excluded. The patients were divided into non-NLND and NLND groups based on examined lymph node (ELN) data. Optimal tumor size cutoff values were defined using X-tile software (version 3.6.1) and divided into the following groups: < 4, 4–10, and ≥ 10 cm (Fig. 2). The primary outcomes were overall survival, defined as the time from diagnosis to death of any cause, and cancer-specific survival, defined as the time from diagnosis to death resulting from the primary STS.

### Statistical analysis

Categorical variates are presented as frequencies and percentages, and continuous variates are presented as the median and interquartile range (IQR). For categorical variables, the Chi-square test was used to analyze between-group differences and for continuous variables, the Wilcoxon rank-sum tests was used. For baseline variables, univariate analysis using the Kaplan–Meier curve and the log-rank test was performed. When the *P* value was < 0.1, variables were included in multivariate Cox regression analysis to identify independent prognostic factors and estimate the hazard ratio (HR) and 95% confidence interval (CI) of each covariate. Risk factors for LNM in STS of the head, neck, and extremities were identified using univariate and multivariate logistic regression analyses. Then, we further analyzed the effect of NLND on the prognosis of patients with STS. Taking non-cancer-specific death as a competing risk to cancer-specific death, cumulative incidence function curves for patients with NLND were plotted, and Gray’s test was performed to compare fatality rates. Multivariate analysis was performed on the cohort data to identify independent prognostic factors by using the Cox proportional hazards regression model and the Fine and Gray’s regression model. In the Cox analysis, patients who were alive at the last follow-up were considered as censored cases, while in the Fine and Gray’s regression model, non-cancer-specific death was considered a competing risk, and we used the sub-distribution hazards ratio (SHR) was used to represent the contribution of each variable to cancer-specific death. The effect of NLND on patient prognosis was also investigated. To eliminate the impact of other factors and minimize the selection bias between the NLND and non-NLND groups, patients from each group were matched 1:1 using propensity score matching (PSM). Chi-square was used to compare the clinicopathological features of the NLND and non-NLND groups. Variables that differed between the two groups as well as those thought to influence treatment for negative lymph nodes were included in the matching analysis. After PSM, Kaplan–Meier and cumulative incidence function curves were created. All statistical analyses were performed using R software (version 3.6.2). The R “survival” package was used for the Kaplan–Meier curve and the Cox regression analysis, the “Matchit” package was used for the PSM analysis, and the “timereg” and “cmprsk” packages were used for the competing risk analysis. A two-sided P value of < 0.05 was considered statistically significant.

## Results

### Demographics and clinical characteristics of patients with STS of the head, neck, and extremities

**Fig. 3:**
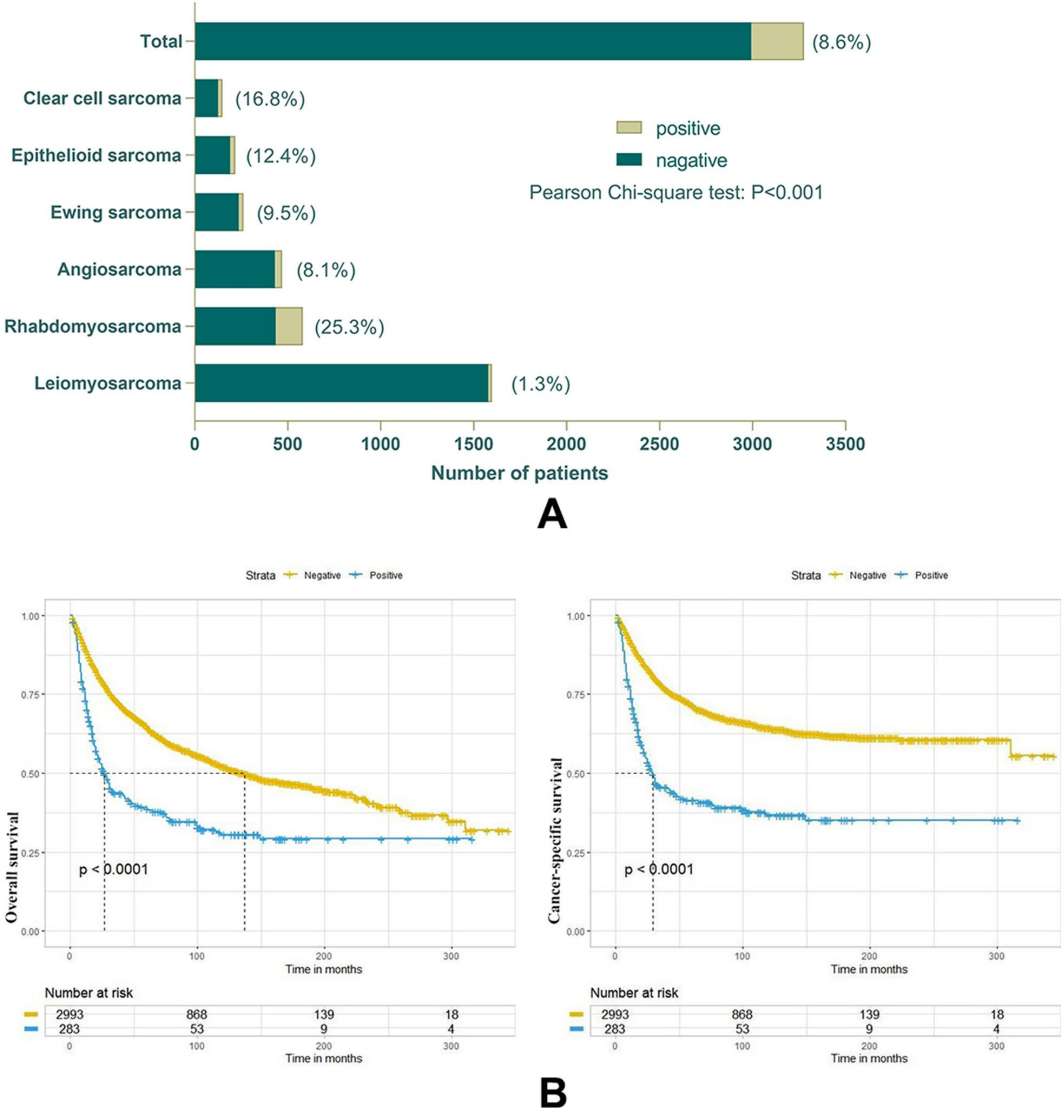
LNM rates for different histological subtypes of STS and the Kaplan–Meier survival curve analysis of the impact of LNM on the prognosis of STS: A, LNM rates for different tumors; B, overall survival and cancer-specific survival of LNM

A total of 3276 eligible cases were included in this study. The median age (IQR range) of the entire cohort population was 53 years (31–69 years). The lymph node positivity rate for the entire cohort was 8.6% and varied significantly between tumor types (Fig. 3A, *P* < 0.001). Lymph node positivity was the highest for rhabdomyosarcoma (25.3%), followed by clear cell sarcoma (16.8%) and epithelioid sarcoma (12.4%), and was the lowest for leiomyosarcoma (1.3%). The cohort was divided into a negative lymph node group (NLN) and a positive lymph node group (PLN). Patients in the PLN group were significantly younger than those in the NLN group [median (IQR), 24 years (10–51 years) vs. 54 years (35–70 years), respectively; *P* < 0.001] (Table 1). In the PLN group, tumors were mostly located in the head, neck, and lower extremities, while in the NLN group, they were primarily found in the lower extremities (73.1% vs. 55.7%, respectively; *P* < 0.001). Rhabdomyosarcoma was predominant in the PLN group (51.9%), while leiomyosarcoma was predominant in the NLN group (52.7%). The rate of NLND was higher in the PLN group than in the NLN group (56.5% vs. 12.6%, respectively; *P* < 0.001). There were also significant differences in race, marital status and tumor grade, stage, size, laterality, and surgery between the two groups (*P* < 0.05). The median survival time was significantly shorter in the PLN than NLN groups [median (IQR), 23 months (11.5–74.5 months) vs. 55 months (22–112 months), respectively; *P* < 0.001].

**Table 1:** Characteristics of enrolled patients divided into lymph node-negative and node-positive groups

Characteristics	Total	Negative	Positive	*p* value^a^
*N* (%)	3276	2993 (91.4%)	283 (8.64%)	
Age, median (IQR^b^)	53.0 [31.0;69.0]	54.0 [35.0;70.0]	24.0 [10.0;51.0]	**< 0.001**
Age, years (%)				**< 0.001**
< 19	537 (16.4%)	411 (13.7%)	126 (44.5%)	
≥ 19	2739 (83.6%)	2582 (86.3%)	157 (55.5%)	
Race (%)				**0.002**
White	2700 (82.4%)	2487 (83.1%)	213 (75.3%)	
Black	338 (10.3%)	293 (9.79%)	45 (15.9%)	
Other^c^	238 (7.26%)	213 (7.12%)	25 (8.83%)	
Sex (%)				0.055
Female	1398 (42.7%)	1293 (43.2%)	105 (37.1%)	
Male	1878 (57.3%)	1700 (56.8%)	178 (62.9%)	
Year of diagnosis (%)				0.664
1988–2005	1286 (39.3%)	1171 (39.1%)	115 (40.6%)	
2006–2015	1990 (60.7%)	1822 (60.9%)	168 (59.4%)	
Site (%)				**< 0.001**
Head, Face, Neck	676 (20.6%)	581 (19.4%)	95 (33.6%)	
Upper limb, shoulder	820 (25.0%)	744 (24.9%)	76 (26.9%)	
Lower limb, hip	1780 (54.3%)	1668 (55.7%)	112 (39.6%)	
Grade (%)				**< 0.001**
I + II	687 (21.0%)	680 (22.7%)	7 (2.47%)	
III + IV	1325 (40.4%)	1216 (40.6%)	109 (38.5%)	
Unknown	1264 (38.6%)	1097 (36.7%)	167 (59.0%)	
Laterality (%)				**< 0.001**
Left	1382 (42.2%)	1285 (42.9%)	97 (34.3%)	
Right	1372 (41.9%)	1255 (41.9%)	117 (41.3%)	
Unknown	522 (15.9%)	453 (15.1%)	69 (24.4%)	
Histology (%)				**< 0.001**
Leiomyosarcoma	1598 (48.8%)	1577 (52.7%)	21 (7.42%)	
Rhabdomyosarcoma	581 (17.7%)	434 (14.5%)	147 (51.9%)	
Angiosarcoma	469 (14.3%)	431 (14.4%)	38 (13.4%)	
Ewing sarcoma	262 (8.00%)	237 (7.92%)	25 (8.83%)	
Epithelioid sarcoma	217 (6.62%)	190 (6.35%)	27 (9.54%)	
Clear cell sarcoma	149 (4.55%)	124 (4.14%)	25 (8.83%)	
Stage (%)				**< 0.001**
Localized	2177 (66.5%)	2177 (72.7%)	0 (0.00%)	
Regional	820 (25.0%)	636 (21.2%)	184 (65.0%)	
Distant	279 (8.52%)	180 (6.01%)	99 (35.0%)	
Surgery (%)				**< 0.001**
No	331 (10.1%)	237 (7.92%)	94 (33.2%)	
Yes	2945 (89.9%)	2756 (92.1%)	189 (66.8%)	
Lymph nodes dissection (%)				**< 0.001**
No	2738 (83.6%)	2615 (87.4%)	123 (43.5%)	
Yes	538 (16.4%)	378 (12.6%)	160 (56.5%)	
Size, cm (%)				**< 0.001**
< 4	1362 (41.6%)	1297 (43.3%)	65 (23.0%)	
4–10	1300 (39.7%)	1163 (38.9%)	137 (48.4%)	
≥ 10	614 (18.7%)	533 (17.8%)	81 (28.6%)	
Marital status (%)				**< 0.001**
Married	1531 (46.7%)	1455 (48.6%)	76 (26.9%)	
Never married	1123 (34.3%)	939 (31.4%)	184 (65.0%)	
Divorced/Widowed/Separated	503 (15.4%)	484 (16.2%)	19 (6.71%)	
Unknown	119 (3.63%)	115 (3.84%)	4 (1.41%)	
Survival months, median (IQR)	52.0 [21.0;110]	55.0 [22.0;112]	23.0 [11.5;74.5]	**< 0.001**

^a^Chi-square test, bold values mean *p* < 0.05, which represent statistically significant

^b^Interquartile range

^c^American Indian/Alaska Native, Asian/Pacific Islander

### Risk factors for LNM of STS in head and neck and extremities

Our results suggested that patients with LNM have a poorer prognosis (Fig. 3B), reinforcing the need to identify risk factors. Univariate logistics and multivariate cox regression analyses were used on the target cohort to identify risk factors for LNM. Variables with *P* values < 0.1 in the univariate analysis were included in the multivariate analysis to adjust for potential confounding (Table 2). The results indicated that patients who were male [odds ratio (OR): 1.291, 95% CI 1.012–1.646; *P* = 0.040], had grade III + IV (OR: 3.930, 95% CI 1.805–8.554; *P* = 0.001) had grade unknown (OR: 5.033, 95%CI 2.325–10.895; *P* < 0.001) tumors, a diagnosis of rhabdomyosarcoma (OR: 9.598, 95%CI 5.719–16.110; *P* < 0.001), angiosarcoma (OR: 5.459, 95% CI 3.163–9.419; *P* < 0.001), Ewing sarcoma (OR: 4.026, 95% CI 2.205–7.351; *P* < 0.001), epithelioid sarcoma (OR: 7.965, 95% CI 4.435–14.307; *P* < 0.001), or clear cell sarcoma (OR: 11.587, 95% CI 6.39–20.989; *P* < 0.001), or tumors that were 4–10 cm (OR: 2.080, 95% CI 1.521–2.845; *P* < 0.001) or ≥ 10 cm (OR: 4.676, 95% CI 3.250–6.728; *P* < 0.001) in size had a higher risk of LNM. Patients with tumors on the upper (OR: 0.549, 95% CI 0.345–0.876; *P* = 0.012) or lower limbs (OR: 0.394, 95% CI 0.249–0.626; *P* < 0.001) had a lower risk of LNM than those with tumors on the head, face, and neck.

**Table 2:** Univariate logistics and multivariate Cox regression analysis for risk factors of lymph node metastasis

Variables	Univariate analysis		Multivariate analysis	
	OR (95% CI)	*p* value	OR (95% CI)	*p* value
Age, years				
< 19	Reference		Reference	
≥ 19	0.198 (0.153–0.256)	**< 0.001**	0.927 (0.681–1.263)	0.633
Race				
White	Reference		Reference	
Black	1.793 (1.272–2.529)	**0.001**	1.293 (0.933–1.791)	0.123
Other	1.37 (0.885–2.122)	0.158	1.044 (0.687–1.585)	0.841
Sex				
Female	Reference		Reference	
Male	1.289 (1.002–1.659)	**0.048**	1.291 (1.012–1.646)	**0.040**
Site				
Head, Face, Neck	Reference		Reference	
Upper limb, shoulder	0.625 (0.453–0.861)	**0.004**	0.549 (0.345–0.876)	**0.012**
Lower limb, hip	0.411 (0.308–0.548)	**< 0.001**	0.394 (0.249–0.626)	**< 0.001**
Grade				
I + II	Reference		Reference	
III + IV	8.708 (4.032–18.806)	**< 0.001**	3.93 (1.805–8.554)	**0.001**
Unknown	14.788 (6.901–31.691)	**< 0.001**	5.033 (2.325–10.895)	** < 0.001**
Laterality				
Left	Reference		Reference	
Right	1.235 (0.933–1.634)	0.14	1.196 (0.912–1.57)	0.196
Unknown	2.018 (1.455–2.798)	** < 0.001**	0.792 (0.494–1.27)	0.334
Histology				
Leiomyosarcoma	Reference		Reference	
Rhabdomyosarcoma	25.435 (15.906–40.673)	**< 0.001**	9.598 (5.719–16.11)	**< 0.001**
Angiosarcoma	6.621 (3.845–11.401)	**< 0.001**	5.459 (3.163–9.419)	**< 0.001**
Ewing sarcoma	7.921 (4.365–14.377)	**< 0.001**	4.026 (2.205–7.351)	**< 0.001**
Epithelioid sarcoma	10.671 (5.917–19.247)	**< 0.001**	7.965 (4.435–14.307)	**< 0.001**
Clear cell sarcoma	15.14 (8.241–27.817)	**< 0.001**	11.587 (6.397–20.989)	**< 0.001**
Size, cm				
< 4	Reference		Reference	
4–10	2.351 (1.732–3.191)	**< 0.001**	2.08 (1.521–2.845)	**< 0.001**
≥ 10	3.032 (2.155–4.267)	**< 0.001**	4.676 (3.25–6.728)	**< 0.001**

*OR* odds ratio, *CI* confidence interval

Bold values mean *p* value < 0.05, which represent statistically significant

### Prognostic factors for patients with STS without LNM in the head, neck, and extremities

The 2756 patients without LNM were included in the survival analysis. To adjust for potential confounding, multivariate Cox regression for cancer-specific survival was conducted by including all possible prognostic factors. Age, marital status and tumor grade, histology, stage, and size were independent prognostic factors for cancer-specific survival (Additional file 1: Table S1). In the Cox proportional hazards regression analysis for cancer-specific survival, patients dying for other reasons are usually censured because death from other causes can prevent the occurrence of target events. Considering death from other causes as competing risk, Fine and Gray’s regression analysis was also conducted. Factors associated with cancer-specific survival after controlling for competing risks included age, marital status and tumor histology, size, grade, and stage. Patients ≥ 19 years of age (sHR, 1.876, 95% CI 1.341–2.625; *P* < 0.001), with tumor grade III + IV (sHR: 1.771, 95% CI 1.422–2.206; *P* < 0.001), other (sHR: 1.400, 95%CI 1.100–1.783; *P* = 0.01), a diagnosis of rhabdomyosarcoma (sHR: 1.387, 95%CI 1.080–1.779; *P* = 0.01), angiosarcoma (sHR: 1.817, 95% CI 1.477–2.237; *P* < 0.001), epithelioid sarcoma (sHR: 1.463, 95% CI 1.029–2.080; *P* = 0.03), or clear cell sarcoma (sHR: 2.321, 95% CI 1.616–3.333; *P* < 0.001), regional (sHR, 1.452, 95% CI 1.220–1.728; *P* < 0.001) or distant (sHR, 4.428, 95% CI 3.356–5.843; *P* < 0.001) state tumors, tumors that were 4–10 cm (sHR: 2.655, 95% CI 2.174–3.243; *P* < 0.001) or ≥ 10 cm (sHR: 4.656, 95% CI 3.699–5.861; *P* < 0.001) in size, or a record of being Divorced/Widowed/Separated (sHR: 1.554, 95% CI 1.272–1.897; *P* < 0.001) were associated with increased risk of cancer-specific death. However, NLND was not associated with cancer-specific mortality (sHR: 0.838, 95% CI 0.662–1.061; *P* = 0.14). Sex, race, year of diagnosis, tumor site, and laterality had no statistical effect on cancer-specific death (Table 3).

**Table 3:** Multiple competing risk regression analysis of soft tissue sarcoma of the head, neck, and extremities without lymph node metastasis

Variables	sHR (95%CI)	*p* value
Age, years		
< 19	Reference	
≥ 19	1.876 (1.341–2.625)	**< 0.001**
Sex		
Female	Reference	
Male	0.96 (0.822–1.120)	0.60
Race		
White	Reference	
Black	1.098 (0.865–1.393)	0.44
Other	1.031 (0.759–1.400)	0.84
Year of diagnosis		
1988–2005	Reference	
2006–2015	0.917 (0.788–1.066)	0.26
Site		
Head, Face, Neck	Reference	
Upper limb, shoulder	0.817 (0.475–1.406)	0.47
Lower limb, hip	0.926 (0.547–1.568)	0.77
Grade		
I + II	Reference	
III + IV	1.771 (1.422–2.206)	**< 0.001**
Other	1.400 (1.100–1.783)	**0.01**
Laterality		
Left	Reference	
Right	1.100 (0.932–1.300)	0.26
Unknown	1.581 (0.916–2.729)	0.10
Histology		
Leiomyosarcoma	Reference	
Rhabdomyosarcoma	1.387 (1.080–1.779)	**0.01**
Angiosarcoma	1.817 (1.477–2.237)	**< 0.001**
Ewing sarcoma	0.807 (0.553–1.178)	0.27
Epithelioid sarcoma	1.463 (1.029–2.080)	**0.03**
Clear cell sarcoma	2.321 (1.616–3.333)	**< 0.001**
Stage		
Localized	Reference	
Regional	1.452 (1.220–1.728)	**< 0.001**
Distant	4.428 (3.356–5.843)	**< 0.001**
Negative lymph nodes dissection		
No	Reference	
Yes	0.838 (0.662–1.061)	0.14
Size, cm		
< 4	Reference	
4–10	2.655 (2.174–3.243)	**< 0.001**
≥ 10	4.656 (3.699–5.861)	**< 0.001**
Marital status		
Married	Reference	
Never married	1.116 (0.912–1.366)	0.29
Divorced/widowed/separated	1.554 (1.272–1.897)	**< 0.001**
Unknown	1.170 (0.746–1.837)	0.49

*sHR* sub-distribution hazard ratio

The values in bold indicate that the *p* value < 0.05, and the results are statistically significant

### The effect of NLND on the prognosis of patients with different soft tissue sarcomas

**Fig. 4:**
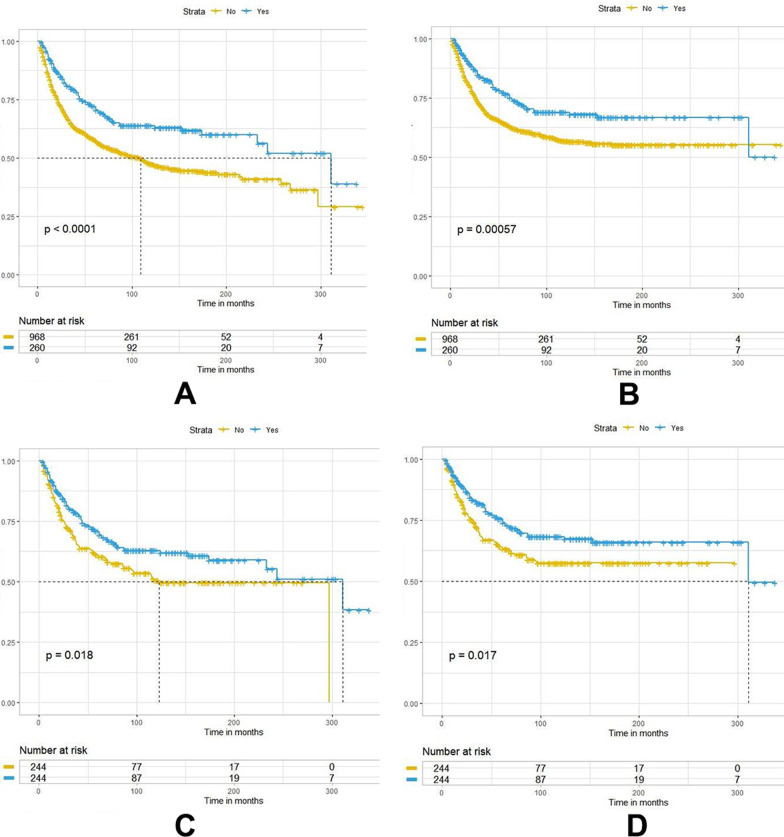
Kaplan–Meier survival curves for overall survival and cancer-specific survival of five STS subtypes with a high risk of LNM between the NLND and non-NLND groups: **A–B** raw cohort; **C**–**D** PSM cohort

**Fig. 5:**
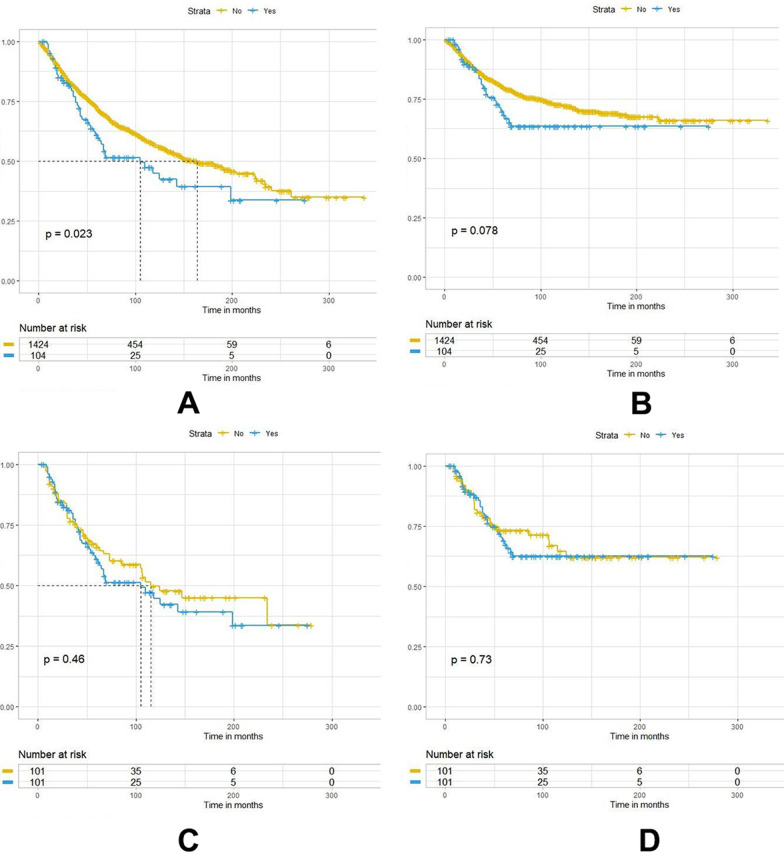
Kaplan–Meier survival curves for overall survival and cancer-specific survival between the NLND group and non-NLND group with leiomyosarcoma: **A**–**B** raw cohort; **C–D** PSM cohort

**Fig. 6:**
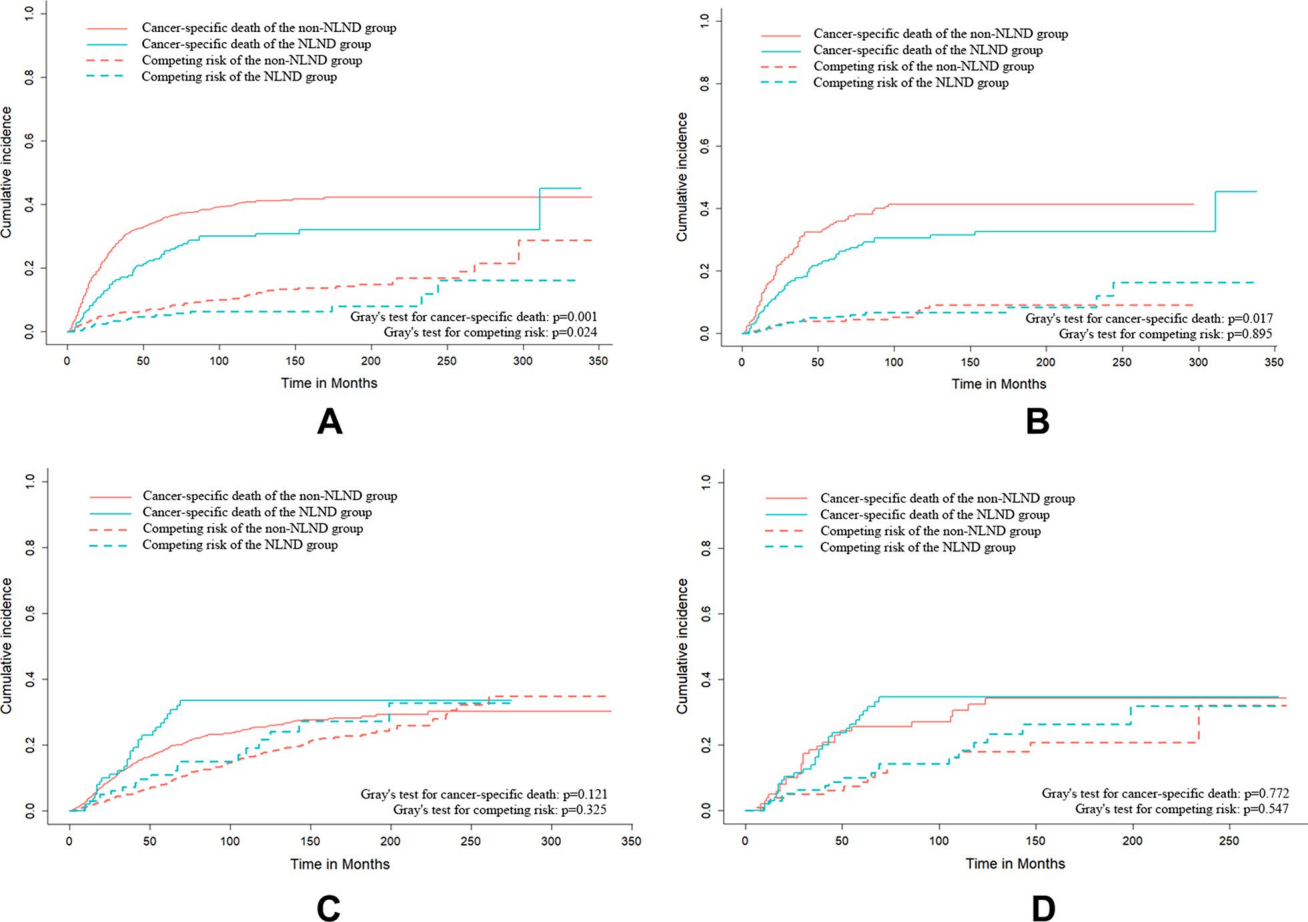
Cumulative incidence of cancer-specific death and competitive risk between the NLND and non-NLND groups of patients with five STS subtypes with a high risk of LNM (**A**, **B**) or leiomyosarcoma (**C**, **D**): A/C, raw cohort; B/D, PSM cohort

The effect of NLND on the prognosis of patients with different histological STS subtypes was assessed. NLND was an independent risk factor for the prognosis of five subtypes of STS at high risk for LNM: rhabdomyosarcomas, angiosarcomas, Ewing sarcomas, epithelioid sarcomas, and clear cell sarcomas. In all cases, the NLND group had significantly higher overall and cancer-specific survival rates (Fig. 4A, B). Cumulative incidence function curves were also plotted to compare the differences in cancer-specific mortality between the two groups. As shown in Fig. 6A, the cumulative incidence of cancer-specific death was lower in the NLND group than in the non-NLND group (Gray’s test, *P* = 0.001). However, NLND did not improve cancer-specific survival in patients with leiomyosarcoma (Fig. 5B). To further clarify the impact of NLND on patient prognosis, PSM analysis was performed. First, Chi-square was used to compare the baseline characteristics of patients in the NLND and non-NLND groups. Results showed significant differences in age, marital status and tumor site, laterality, histology, stage, and size, between the two groups (*p* < 0.05, Table 4). The covariables were then matched to create a new cohort. After PSM, the covariates were balanced between the two groups, and the results further confirmed that NLND significantly improved both overall (HR: 0.718, 95%CI 0.535–0.962; *P* = 0.026) and cancer-specific survival (HR: 0.699, 95%CI 0.506–0.967; *P* = 0.031) (Table 5 and Fig. 4C, D). Patients in the NLND group had significantly lower cancer-specific mortality (Gray’s test, *P* = 0.017) (Fig. 6B), and significantly longer median survival time than patients in the non-NLND group, both before (median: 69 vs. 47 months, respectively; *p* < 0.001) and after PSM (median: 69.5 vs. 57.5 months, respectively; *p* = 0.080). However, NLND did not improve overall survival (*P* = 0.46) or reduce cancer-specific mortality (Gray’s test, *P* = 0.772) of patients with leiomyosarcoma (Figs. 5C, D, 6D).

**Table 4:** Clinical characteristics of five types of soft tissue sarcomas with a high risk of lymph node metastasis, before and after propensity score matching (PSM)

Variables	Lymph node treatment group in raw data			Lymph node treatment group after PSM		
	Non-NLND (*n* = 968)	NLND (*n* = 260)	*P* value	Non-NLND (*n* = 244)	NLND (*n* = 244)	*P* value
Age, median (IQR)	46.0 [22.0;68.0]	26.0 [12.0;47.2]	< 0.001	34.5 [13.0;57.2]	29.0 [12.0;48.2]	0.017
Age, years			**< 0.001**			0.703
< 19	196 (20.2%)	97 (37.3%)		81 (33.2%)	86 (35.2%)	
≥ 19	772 (79.8%)	163 (62.7%)		163 (66.8%)	158 (64.8%)	
Race			0.468			0.039
White	772 (79.8%)	215 (82.7%)		180 (73.8%)	203 (83.2%)	
Black	107 (11.1%)	27 (10.4%)		36 (14.8%)	24 (9.84%)	
Other	89 (9.19%)	18 (6.92%)		28 (11.5%)	17 (6.97%)	
Sex			0.173			0.069
Female	421 (43.5%)	126 (48.5%)		99 (40.6%)	120 (49.2%)	
Male	547 (56.5%)	134 (51.5%)		145 (59.4%)	124 (50.8%)	
Year of diagnosis			0.231			1
1988–2005	364 (37.6%)	109 (41.9%)		105 (43.0%)	105 (43.0%)	
2006–2015	604 (62.4%)	151 (58.1%)		139 (57.0%)	139 (57.0%)	
Site			**< 0.001**			0.711
Head, Face, Neck	260 (26.9%)	52 (20.0%)		59 (24.2%)	52 (21.3%)	
Upper limb, shoulder	200 (20.7%)	97 (37.3%)		87 (35.7%)	87 (35.7%)	
Lower limb, hip	508 (52.5%)	111 (42.7%)		98 (40.2%)	105 (43.0%)	
Grade			0.127			0.899
I + II	114 (11.8%)	25 (9.62%)		22 (9.02%)	25 (10.2%)	
III + IV	408 (42.1%)	97 (37.3%)		94 (38.5%)	93 (38.1%)	
Unknown	446 (46.1%)	138 (53.1%)		128 (52.5%)	126 (51.6%)	
Laterality			**0.022**			0.9
Left	364 (37.6%)	119 (45.8%)		111 (45.5%)	106 (43.4%)	
Right	399 (41.2%)	102 (39.2%)		95 (38.9%)	99 (40.6%)	
Unknown	205 (21.2%)	39 (15.0%)		38 (15.6%)	39 (16.0%)	
Histology			**< 0.001**			0.949
Rhabdomyosarcoma	255 (26.3%)	78 (30.0%)		73 (29.9%)	78 (32.0%)	
Angiosarcoma	344 (35.5%)	42 (16.2%)		44 (18.0%)	42 (17.2%)	
Ewing sarcoma	184 (19.0%)	19 (7.31%)		18 (7.38%)	19 (7.79%)	
Epithelioid sarcoma	109 (11.3%)	75 (28.8%)		69 (28.3%)	62 (25.4%)	
Clear cell sarcoma	76 (7.85%)	46 (17.7%)		40 (16.4%)	43 (17.6%)	
Stage			**0.05**			0.661
Localized	663 (68.5%)	171 (65.8%)		169 (69.3%)	160 (65.6%)	
Regional	238 (24.6%)	79 (30.4%)		65 (26.6%)	74 (30.3%)	
Distant	67 (6.92%)	10 (3.85%)		10 (4.10%)	10 (4.10%)	
Size, cm			**0.006**			0.597
< 4	368 (38.0%)	121 (46.5%)		109 (44.7%)	114 (46.7%)	
4–10	407 (42.0%)	107 (41.2%)		109 (44.7%)	99 (40.6%)	
≥ 10	193 (19.9%)	32 (12.3%)		26 (10.7%)	31 (12.7%)	
Marita status			**< 0.001**			0.858
Married	407 (42.0%)	70 (26.9%)		76 (31.1%)	69 (28.3%)	
Never married	404 (41.7%)	152 (58.5%)		134 (54.9%)	139 (57.0%)	
Divorced/Widowed/Separated	138 (14.3%)	29 (11.2%)		29 (11.9%)	29 (11.9%)	
Unknown	19 (1.96%)	9 (3.46%)		5 (2.05%)	7 (2.87%)	
Survival months (median, IQR)	47.0 [17.0;108]	69.0 [26.8;136]	**< 0.001**	57.5 [21.0;120]	69.5 [26.8;136]	0.08

*NLND* negative lymph node dissection

The values in bold indicate that the *p* value < 0.05, and the results are statistically significant

**Table 5:** Multivariate Cox analysis of OS and CSS of five types of soft tissue sarcomas with a high risk of lymph node metastasis after propensity score matching (PSM)

Variables	Overall survival		Cancer-specific survival	
	HR(95%CI)	*P*	HR(95%CI)	*P*
Age, years				
< 19	Reference		Reference	
≥ 19	3.218 (1.868–5.543)	**< 0.001**	2.702 (1.49–4.901)	**0.001**
Year of diagnosis				
1988–2005	Reference			
2006–2015	1.089 (0.805–1.472)	0.581		
Site				
Head, Face, Neck	Reference		Reference	
Upper limb, shoulder	0.577 (0.3–1.108)	0.099	0.695 (0.324–1.49)	0.35
Lower limb, hip	0.691 (0.367–1.299)	0.251	0.789 (0.378–1.649)	0.529
Grade				
I + II	Reference		Reference	
III + IV	1.052 (0.613–1.805)	0.854	1.598 (0.825–3.096)	0.165
Unknown	1.19 (0.695–2.037)	0.526	1.704 (0.872–3.328)	0.119
Laterality				
Left				
Right	1.047 (0.752–1.457)	0.786	1.024 (0.712–1.474)	0.896
Unknown	0.944 (0.481–1.854)	0.868	1.005 (0.457–2.212)	0.989
Histology				
Rhabdomyosarcoma	Reference		Reference	
Angiosarcoma	0.942 (0.574–1.545)	0.814	1.258 (0.724–2.187)	0.415
Ewing sarcoma	0.397 (0.187–0.845)	**0.02**	0.53 (0.236–1.187)	0.123
Epithelioid sarcoma	0.692 (0.415–1.154)	0.158	0.825 (0.464–1.466)	0.512
Clear cell sarcoma	0.785 (0.455–1.355)	0.385	1.08 (0.593–1.969)	0.801
Stage				
Localized	Reference		Reference	
Regional	1.501 (1.078–2.09)	**0.02**	1.689 (1.181–2.415)	**0.004**
Distant	3.336 (1.776–6.266)	**< 0.001**	4.308 (2.259–8.217)	**< 0.001**
NLND				
No	Reference		Reference	
Yes	0.718 (0.535–0.962)	**0.03**	0.699 (0.506–0.967)	**0.031**
Size, cm				
< 4	Reference		Reference	
4–10	1.816 (1.26–2.616)	**0**	2.3 (1.513–3.496)	**< 0.001**
≥ 10	2.708 (1.636–4.482)	**< 0.001**	3.266 (1.857–5.745)	**< 0.001**
Marital status				
Married	Reference		Reference	
Never married	0.949 (0.633–1.422)	0.799	0.976 (0.627–1.52)	0.915
Divorced/widowed/separated	1.971 (1.279–3.037)	**0**	1.832 (1.13–2.972)	**0.014**
Unknown	1.077 (0.465–2.491)	0.863	1.046 (0.39–2.804)	0.929

The values in bold indicate that the *p* value < 0.05, and the results are statistically significant

## Discussion

The prognosis for STS patients with metastasis is poor. The primary STS metastasis sites include the lung and bone, with LNM being relatively rare. However, according to the latest AJCC staging guidelines, sarcomas of the trunk and extremities that involve the lymph nodes are defined as stage IV disease [1]. Thus, it is critical to understand the risk factors and prognosis of STS patients with LNM. To date, most studies have assessed all STS together or site- and patient-specific STS. For example, Gusho et al. studied the LNM rate and prognosis of all STS of the extremities [11]. Sherman et al. studied the LNM rate and predictors of adult STS of extremities [15]. The current study included six types of STS of the head, neck, and extremities that are associated with the highest risk of LNM. The prognosis of 2756 STS patients without LNM who received surgical treatment was quantified. This study also clarified the impact of NLND on STS patient prognosis.

In our cohort, rhabdomyosarcoma (25.3%), clear cell sarcoma (16.8%), and epithelioid sarcoma (12.4%) patients had the highest rates of LNM, while leiomyosarcoma patients had the lowest rate of LNM (1.3%). This is consistent with previously reported rates of lymph node-positivity in different STS types (26.7% for rhabdomyosarcoma, 16–18.8% for clear cell sarcoma, and 13–14.5% for epithelioid sarcoma) from several large cohort studies [8, 11, 16]. In children and adolescents (< 19 years of age), rhabdomyosarcoma had the highest lymph node-positivity rate, while in adults (≥ 19 years), clear cell sarcoma had the highest positivity rate. These results are similar to those found in previous studies [8, 15]. This may be the result of differences in the histological subtypes of STS by age. Indeed, rhabdomyosarcoma was the most common STS in children and adolescents and accounts for one-half of pediatric STS [17]. Independent risk factors for LNM were identified using the multivariate Cox proportional risk model. Patients who were male or had STS in the head and neck, high-grade (III + IV), tumors > 4 cm in size, or non-leiomyosarcomas were more likely to have LNM. Several studies have reported similar results. Miccio et al. found that patients with high-grade STS and histology including clear cell, angiosarcoma, rhabdomyosarcoma, and epithelioid (CARE) sarcomas were associated with LNM [7]. Behranwala et al. examined 2127 STS patients and found a 70% association between high-grade tumors and lymph nodes spread, with LNM more likely appearing in the proximal location of the sarcoma [6]. Sherman et al. analyzed 27,536 extremity soft tissue sarcoma (ESTS) patients from the National Cancer Data Base (2000–2009) and found that histologic subtype, tumor size, and tumor grade were risk factors for LNM [15]. Other studies have shown that LNM also correlates with age and primary site [10, 18], but none have reported an association between gender and LNM. Our results suggest that males are at higher risk of LNM than females (OR: 1.291, 95% CI 1.012–1.646; *P* = 0.040). Studies show that males have a higher predisposition for STS than females, but the extent to which gender affects LNM has not been established [19].

This study also performed a prognostic analysis of 2756 patients without LNM who had undergone surgery. Age, grade, stage, size, histology and marital status were found to be independent prognostic factors for cancer-specific survival. This result is similar to previous studies. A study with patient data also from the SEER database showed that for the historically high-risk extremity, STS, age, grade, size, surgery, and regional lymph node status were independent disease-specific prognostic factors [11]. Another study found that for epithelial sarcoma, tumor site was a prognostic factor for event-free survival and overall survival, and extremities site had a better prognosis than proximal-type variant [20]. Understanding these STS characteristics can inform clinical counseling and personalized treatment for patients.

The current study further investigated the association between NLND and disease prognosis. After PSM, we found that NLND was an independent prognosis factor for patients with a high risk of LNM such as rhabdomyosarcomas, angiosarcomas, Ewing sarcomas, epithelioid sarcomas, and clear cell sarcomas. Surprisingly, NLND did not improve the prognosis of leiomyosarcoma before and after PSM. Further analysis showed that patients with leiomyosarcoma were older than the rest five types of sarcomas (median age: 61 vs. 41 years, respectively; *P* < 0.001), and only a small proportion of leiomyosarcoma patients (6.81%) received NLND than those with other tumors (21.2%). Unfortunately, no information about why these patients did not receive a lymph node dissection is available in the SEER database. This may be because these patients are older or have comorbidities or treatment contraindications. In addition, the SEER database does not record information about adjuvant treatments such as radiotherapy and chemotherapy. The absence of these data may affect the accuracy of the results and cause confounding bias so further clinical validation is needed.

Currently, there is no consensus guidelines on how to assess lymph node status in high-risk STS patients. Common strategies for lymph node status assessment include physical examination, ultrasound, CT, MRI, and sentinel lymph node biopsy (SLNB). Radical lymphadenectomy or systemic chemotherapy are often recommended for the treatment of regional lymph node metastases, but treatment modalities are mostly clinician- and center-dependent [19]. Some studies have reported the prognosis of lymph node examination/dissection for STS with LNM, but the result remains controversial. Al-Refaie et al. suggest that regional lymph node dissection may prolong survival time [21]. Ecker et al. support regional lymph node examination for patients with epithelioid and possibly clear cell sarcoma [22]. Brady et al. found that lymph node sampling was associated with improved disease-specific survival in patients with extremity rhabdomyosarcoma (64% vs. 49%, *P* = 0.005) [23]. Riad et al. showed that resection of involved lymph nodes had an estimated 5-year survival of 57%, whereas nine patients treated without surgery all died within 30 months [24]. In another study, NLND was proved to be an independent risk factor for cancer-specific survival of non-metastatic colorectal sarcomas patients [25]. However, some studies found that lymph node examination/dissection had no impact on prognosis. One study of epithelioid sarcomas found that lymphadenectomy did not improve the overall survival of patients with LNM [26]. Another study found that resection of the metastatic lymph node had better survival at 1.5 years, but did not improve the long-term survival of patients with STS [9]. Some studies argued that the management of positive lymph nodes remains uncertain and that more research is required to assess the impact of lymphadenectomy on the overall survival of STS patients with LNM [3, 10]. The current study suggests that NLND is an appropriate treatment for specific STS patients such as those at high risk for LNM. Nevertheless, we still recommend that patients' treatment decisions should be based on the clinical reality of the patient, because lymph node dissection may have some acute and chronic complications, such as lymphorrhea, chylous ascites, seroma, delayed wound healing, and chronic lymphedema [27].

This study has some limitations which need to be considered. First, this is a retrospective study and may have inherent limitations, so the results must be validated using prospective studies. Second, the SEER database does not record detailed information about chemotherapy, radiotherapy, comorbidities, complications, and recurrence, which may have a potential impact on the results. Despite these limitations, our findings are of significance.

## Conclusion

This study identified the rate of LNM in patients with six STS subtypes of the head, neck, and extremities. In addition, risk factors for LNM and the prognostic factors for STS patients without LNM were further clarified. Most importantly, our study suggests that prophylactic lymph node dissection was necessary and had a clinically beneficial for STS patients at high risk for LNM in the head, neck, and extremities. However, for leiomyosarcoma, NLND did not improve the prognosis and prophylactic lymph node dissection needs to be more carefully evaluated.

## Supplementary Information

**Additional file 1: Table S1.** Univariate logistics and Multivariate Cox analysis of cancer-specific survival for 2756 patients without LNM.

## Data Availability

The datasets used and/or analyzed during the current study are available from the corresponding author on reasonable request.

## Abbreviations

LNM: Lymph node metastasis
STS: Soft tissue sarcomas
NLND: Negative lymph node dissection
SEER: Surveillance, epidemiology, and end results
PSM: Propensity score matching analysis
IQR: Interquartile range
HR: Hazard ratio
CI: Confidence interval
SHR: Sub-distribution hazards ratio
PLN: Positive lymph node group
